# Asian elephants (*Elephas maximus*) recognise human visual attention from body and face orientation

**DOI:** 10.1038/s41598-025-16994-3

**Published:** 2025-10-02

**Authors:** Hoi-Lam Jim, Shinya Yamamoto, Pakkanut Bansiddhi, Joshua M. Plotnik

**Affiliations:** 1 Institute for the Future of Human Society, Kyoto University, Kyoto, Japan; 2 Japan Society for the Promotion of Science, Tokyo, Japan; 3 Faculty of Veterinary Medicine, Chiang Mai University, Chiang Mai, Thailand; 4 Center of Elephant and Wildlife Health, Chiang Mai University Animal Hospital, Chiang Mai, Thailand; 5 Department of Psychology, Hunter College, City University of New York, New York, NY USA; 6 Department of Psychology, The Graduate Center, City University of New York, New York, NY USA

**Keywords:** Elephants, Social cognition, Attentional state, Psychology, Animal behaviour

## Abstract

Visual attention has mostly been studied in primarily visual species, such as nonhuman primates. Although elephants rely more on acoustic and olfactory cues, they also use visual displays and gestures to communicate. Smet and Byrne (2014) showed that African savanna elephants (*Loxodonta africana*) recognise human visual attention based on face and body orientation, but this has not been investigated in Asian elephants (*Elephas maximus*). We tested ten captive female elephants in Thailand and analysed the frequency of experimenter-directed signals in a food-requesting task based on the experimenter’s body and face orientation. Elephants gestured most when both the experimenter’s body and face were oriented towards them, and body orientation appeared to be a stronger visual cue than face orientation, but this effect was only observed when her face was also oriented towards the elephant. This suggests that elephants are not sensitive to face or body orientation alone and rely on a combination of body and face cues to recognise human visual attention. These findings suggest that Asian elephants understand the importance of visual attention for effective communication, contributing to our understanding of cognitive abilities across the elephant taxon and visual attention in animals.

**Supplementary Information:**

The online version contains supplementary material available at 10.1038/s41598-025-16994-3.

## Introduction

Communication can be defined as the transfer of information from a signaller to a recipient, where the signal conveys information that may influence the behaviour of the receiver (regardless of whether they were the intended recipient) or both participants ^1^. Importantly, the signal must be detected and perceived by the recipient for effective communication ^2^. Animals can use a variety of signal modalities for communication, such as visual, auditory, or olfactory, and signals can be multimodal. Different species have adapted to use different senses to perceive and interpret the world around them to help them survive in their environment. For example, animals that have evolved a sophisticated visual system, including humans and nonhuman primates ^3,4^, often use visual signals to communicate with each other and use vision to navigate their social world ^5^.

Many studies have shown that nonhuman primates adjust their communicative signals depending on the recipient’s attentional state—they produce significantly more visual gestures when a human is present and when the human’s body and/or face is oriented towards them compared to when it is oriented away. This has been demonstrated in chimpanzees (*Pan troglodytes*) ^6,7^, bonobos (*Pan paniscus*) ^7^, orangutans (*Pongo pygmaeus*) ^7,8^, gorillas (*Gorilla gorilla gorilla*) ^8^, Tonkean macaques (*Macaca tonkeana*) ^9^, rhesus macaques (*Macaca mulatta*) ^10^, and free-ranging bonnet macaques (*Macaca radiata*) ^11^. Kaminski et al. ^7^ suggested that body and face orientation convey different types of information, proposing a "bivariate and hierarchical interpretation" of these cues—specifically, that body orientation signals the experimenter’s disposition to offer food, whereas face orientation indicates their attentional state, as the experimenter only gave food when her body was oriented towards the ape. In a follow-up study, Tempelmann et al. ^12^ tested all four great ape species and controlled for the experimenter’s ability to provide food (i.e., food could be delivered regardless of body orientation). Under these conditions, the dominant effect of body orientation was reduced, and face orientation became the main factor influencing begging behaviour. These findings support the hypothesis that body and face orientation are interpreted hierarchically.

As shown above, previous studies on visual attention have primarily focused on visually oriented animals. However, the evolutionary origins of this communicative ability remain poorly understood, particularly in non-primate, non-visually dominant species. To better understand the factors that have shaped sensitivity to visual communicative signals, it is important to expand research to a broader range of species. Elephants, with their highly social nature and reliance on audition and olfaction over vision—evidenced by the significantly larger brain areas dedicated to these senses compared to the visual cortex ^4^—are an ideal study animal for this purpose because they still use visual displays and gestures to communicate with one another ^14^. In fact, this may be particularly beneficial in their complex fission–fusion societies, where cooperation is essential ^15,16^. For example, Smet and Byrne ^17^ suggested that African savanna elephants (*Loxodonta africana*) use the “periscope-sniff” as an ostensive pointing signal, potentially helping others detect and respond to dangers.

Elephants’ sensitivity to the attentional states of others may extend to humans as well. Captive elephants produced more visual gestures when the experimenter was present and facing them compared to when she was absent ^18^. The elephants also modified their experimenter-directed signals in a food-requesting task based on the experimenter’s face and body orientation, gesturing more when her face was oriented towards them, but only when her body was also turned sideways or towards them ^19^, consistent with Kaminski et al.’s ^7^ findings in apes. It is important to note that the animals in these studies had extensive experience with humans, which could make them more sensitive to human attentional states.

Despite these recent advances in our understanding of African savanna elephants’ socio-cognitive abilities, little is known about this in other elephant species. The three living elephant species—African savanna, African forest (*Loxodonta cyclotis*) and Asian (*Elephas maximus*)—belong to the family Elephantidae, with the lineage of Asian elephants diverging from that of African elephants approximately 5–7 million years ago ^20^. Over this timespan, significant species-level differences in behaviour and ecology may have emerged ^13,21,22^, raising questions about the extent to which cognitive capacities are shared across the elephant taxon. Furthermore, although evolutionary rates may differ, the split between *Elephas* and *Loxodonta* is comparable in timing to the divergence between *Homo* and *Pan* lineages in the family Hominidae ^20,23^. Several global environmental changes were occurring at that time, potentially affecting many lineages and contributing to speciation across diverse mammalian groups ^20^. Therefore, studying closely related species may provide valuable insights into how socio-cognitive abilities evolved in response to varying ecological and social pressures. For example, Asian elephants ^24,25^ and African savanna elephants ^26^ performed similarly on a means-end task, suggesting comparable problem-solving abilities. However, other research has found species-level differences—African savanna elephants can use human pointing cues to locate hidden food ^27,28^, but this ability has not been demonstrated in Asian elephants ^13,29^.

To further investigate potential cognitive similarities between elephant species, we tested Asian elephants’ understanding of human visual attention, closely following the methodology of Smet and Byrne ^19^. Thus, we examined whether Asian elephants adjust the frequency of experimenter-directed signals in a food-requesting task based on the experimenter’s body and/or face orientation, and we hypothesised that they are sensitive to the attentional state of others and use visual gestures as communicative signals. For effective visual communication, the signaller must ensure they are within the recipient’s line of sight. Therefore, we predicted that elephants would gesture more frequently when (1) the experimenter was present compared to when she was absent, and (2) when the experimenter’s face and body were oriented towards them compared to when either was oriented away. Since the surface area of the human body is much larger than that of the face, and elephants’ visual acuity may not be sufficient to detect whether a human’s face is oriented towards or away from them at a distance, we further predicted that (3) body orientation would be a stronger visual cue than face orientation.

## Results

A zero-inflated Poisson GLMM showed that the frequency of head and trunk gestures elephants produced to request food varied significantly across experimental conditions (full-null model comparison: χ^2^ = 32.333, *df* = 4, *p* < 0.001; see Table S1). Gesture frequency significantly decreased across sessions (estimate = -0.318, *SE* = 0.078, χ^2^ = 16.571, *df* = 1, *p* < 0.001), possibly due to fatigue or learning effects; since the experimenter (hereafter ‘E’) always provided food after trials, elephants may have learned that gesturing was unnecessary to receive the reward. Gesture frequency did not change significantly across trials (χ^2^ = 0.070, *df* = 1, *p* = 0.791).

**Fig. 1:**
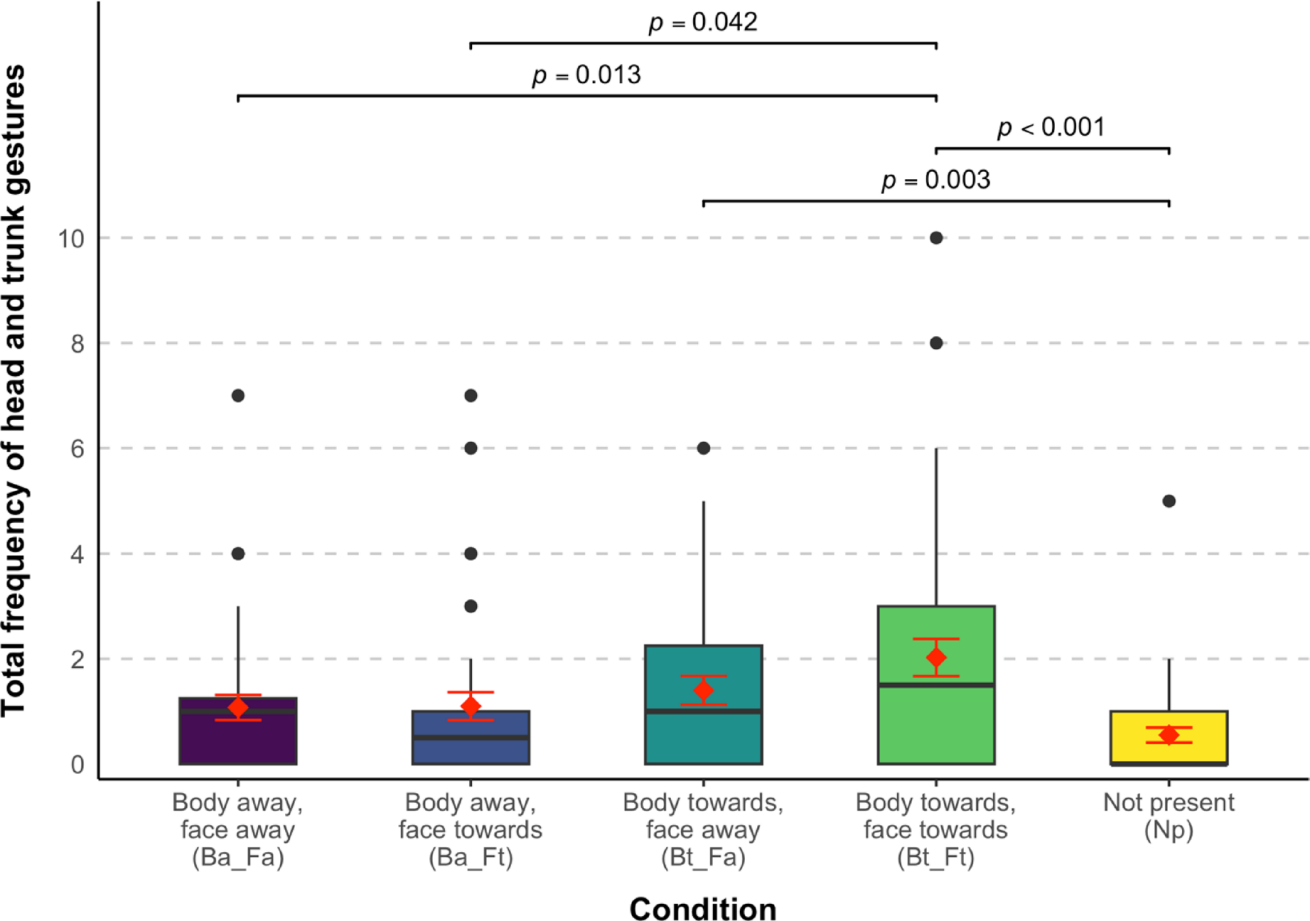
Box plot showing the total frequency of elephants’ head and trunk gestures within a single trial. Black dots represent outliers, red diamonds indicate the mean, and red error bars represent the standard error.

To simplify the presentation of pairwise comparisons, experimental conditions are abbreviated as in the Methods (Experimental design). Elephants gestured significantly more in the two conditions where E’s body was oriented towards them compared to the baseline (Np–Bt_Fa: *p* = 0.003; Np–Bt_Ft: *p* < 0.001; Fig. 1; see Table S2). In contrast, there was no significant difference in gesture frequency between the baseline and the two conditions where E’s body was oriented away (Np–Ba_Fa: *p* = 0.108; Np–Ba_Ft: *p* = 0.058). This suggests that the mere presence of a human did not increase gesturing and that elephants were sensitive to body orientation.

There was a significant difference when comparing the two conditions in which body and face orientation were congruent (Ba_Fa–Bt_Ft: *p* = 0.013), showing that elephants gestured more when both E’s body and face were oriented towards, rather than away from them.

When body orientation was held constant, face orientation did not significantly affect gesture frequency, whether E’s body was oriented away (Ba_Fa–Ba_Ft: *p* = 0.998) or towards the elephants (Bt_Fa–Bt_Ft: *p* = 0.335). This indicates that elephants were not sensitive to face orientation alone.

When face orientation was held constant, elephants gestured significantly more when E’s body was oriented towards them, but only when her face was also directed towards them (Ba_Ft–Bt_Ft: *p* = 0.042). In contrast, there was no significant difference in gesturing when E’s face was oriented away (Ba_Fa–Bt_Fa: *p* = 0.674). This suggests that body orientation alone was insufficient to significantly increase gesturing, and that face orientation may modulate its effect.

## Discussion

The aim of this study was to test whether Asian elephants adjust the frequency of experimenter-directed signals in a food-requesting task based on the experimenter’s body and/or face orientation. We found that (1) the mere presence of a human did not increase gesturing; (2) elephants gestured most when the experimenter’s body and face orientation were both oriented towards them; (3) neither face nor body orientation alone was sufficient to increase gesturing; (4) body orientation appeared to be a stronger visual cue than face orientation, but its effect depended on the face also being oriented towards the elephant; thus, (5) face orientation modulated the influence of body orientation. Taken together, these findings suggest that elephants rely on a combination of body and face cues to recognise human visual attention, with body orientation being the stronger cue when it is congruent with face orientation.

Our results support the hypothesis that Asian elephants are sensitive to the attentional state of others and use visual gestures as communicative signals. The highly significant difference between the ‘body towards, face towards’ and ‘not present’ conditions in the current study aligns with Eleuteri et al.’s ^18^ findings, in which the experimenter’s body and face were always oriented towards the elephants. In contrast, the lack of significant differences between the two ‘body away’ conditions and the ‘not present’ condition suggests that elephants did not gesture more simply due to the presence of a human, contrary to our first prediction. Similarly, Kaminski et al. ^7^ and Smet and Byrne ^19^ found no significant difference in gesture frequency between the ‘body away, face away’ and ‘experimenter absent’ conditions. Taken together, these findings indicate that the presence of a human alone is not sufficient to elicit gesturing; rather, apes and elephants are specifically attuned to visual attentional cues.

We found that elephants gestured most when both the experimenter’s body and face were oriented towards them, supporting our second prediction. They were not sensitive to human face or body orientation in isolation; face orientation alone had no effect, and body orientation only influenced gesturing when the face was also oriented towards the elephant. Thus, we found partial support for our third prediction: body orientation may be a stronger cue than face orientation, but its effect depends on the face also being directed towards the elephant. This supports Kaminski et al.’s ^7^ finding in apes and aligns with their hierarchical, bivariate interpretation that body orientation is prioritised over face orientation in a food-giving context, as it signals the human’s disposition to give food.

Our results do not align with those of Tempelmann et al. ^12^, who found that apes prioritised face orientation when the experimenter’s ability to provide food was controlled. There are two possible explanations for the discrepancy between our findings. First, we did not control for food delivery. In our design, as in Kaminski et al. ^7^, the experimenter only delivered a reward when her body was oriented towards the elephant. This lack of control for food delivery in both body orientation conditions makes it difficult to disentangle whether face orientation has an effect on its own or only when accompanied by a congruent body orientation. Moreover, we did not include a ‘body sideways’ condition as in Smet and Byrne ^19^ due to time constraints; incorporating this condition would have provided another useful control. Future studies should aim to address these limitations by including a ‘body sideways’ condition and controlling for food delivery—if possible, given the husbandry constraints of working with large mammals in captivity—across all body orientations.

Another possible explanation for the discrepancy is that sensitivity to face orientation may be more relevant in primates than elephants. A study on African savanna elephants’ greeting behaviour found that they adjusted their gestures based on a conspecific recipient’s visual attention, preferring visual gestures when the conspecific was looking, and tactile gestures and audible body acts when they were not ^30^. This suggests that elephants are sensitive to conspecific face orientation. However, in our study, it is possible that human faces are too morphologically different from elephant faces for elephants to perceive human attentional states, whereas this may be less of an issue for nonhuman primates.

The finding from this study that body orientation appears to be a stronger visual cue than face orientation when both are ‘towards the elephant’ is consistent with what we know about the relationship between elephant handlers and captive elephants; the latter rely on the former for food, so they may have learned to associate a ‘body towards’ orientation with food availability from a young age. While humans can feed elephants without facing them directly, feeding interactions typically involve both the body and face being oriented towards the elephant for safety reasons. Thus, elephants may prioritise gesturing when both cues are present.

Although we only demonstrated that elephants are sensitive to human body orientation when the face was also oriented towards them, it is possible that body orientation is inherently a more salient visual cue, as the larger surface area of the body likely makes it easier to detect from a distance, especially given elephants’ relatively poor visual acuity ^4,31^. This is further supported by evidence that both Asian ^13,29^ and African savanna elephants ^27^ appear unable to use gaze cues alone to locate food, suggesting that the human face may be insufficiently salient for elephants to perceive without another cue like the body.

Human body orientation may be a particularly relevant cue for wild elephants, as they likely do not approach humans closely enough to perceive their face orientation. They have been shown to discriminate between humans based on olfactory, visual, and auditory cues; African savanna elephants in Amboseli National Park, Kenya, reacted more fearfully to the scent of garments previously worn by a member of the Maasai ethnic group than by a member of the Kamba ethnic group. The elephants also reacted more aggressively to red clothing typically worn by Maasai than to neutral white clothing ^32^. The Maasai are pastoralists and are in significantly more frequent conflict with elephants than the Kamba, who are agriculturalists. These data suggest that elephants perceive the Maasai as a greater threat than the Kamba. In fact, in a separate study, elephants exhibited stronger defensive and investigative behaviours following playbacks of the voices of adult Maasai men compared to those of Kamba men, Maasai women, and Maasai boys ^33^. Although these responses were based on auditory cues, the associated visual characteristics of these groups (i.e., differences in body shape between men, women, and boys) may be perceivable to elephants from a distance. Thus, recognising human body orientation from afar could provide an evolutionary advantage, allowing elephants to assess potential threats more effectively.

This study adds to the literature suggesting that Asian elephants ^24,25^ and African savanna elephants ^26^ share some similarities in cognition. However, these previous studies ^24–26^ examined means-to-end problem-solving, which differs from the focus of the present study. Our study is more closely aligned with research on the following of human pointing cues, as both involve visual cue perception, but conflicting results have been found between the two species ^13,27–29^. We kept our methodology as consistent as possible with that of Smet and Byrne ^19^, and the elephants in both studies had similar rearing histories, having spent most or all of their lives in captivity and being accustomed to close human interaction. Further research is needed to determine whether discrepancies in elephants’ socio-cognitive abilities reflect ecological differences or are influenced by captivity and human exposure ^13^.

There are several limitations of this study that should be acknowledged. Sampling biases—such as rearing history described above—may affect the reproducibility and generalisability of the findings ^34,35^. Some elephants in this study had prior experience with cognitive research, while others participated in another experiment for the first time simultaneously (see Table S3). Additionally, we only tested female elephants that were available and with whom it was safe to work. Like some previous studies on nonhuman primates ^6–10,12^ and elephants ^18,19^ investigating sensitivity to human attentional states, our study involved enculturated individuals with extensive human experience, which may limit the ecological validity of the results. Finally, our small sample size means that individual variation in behaviour and cognition could affect the generalisability of our results.

In conclusion, this study is the first to demonstrate that Asian elephants are sensitive to human attentional states. While body orientation appeared to be a stronger visual cue than face orientation, this effect was only observed when the face was also oriented towards the elephant. This research contributes to the literature on elephant cognition and adds to the growing evidence for convergent evolution of socio-cognitive abilities across evolutionarily distant taxa, including elephants and great apes.

## Methods

Our hypotheses, predictions, study design, and the behavioural and statistical analysis plan were pre-registered (https://aspredicted.org/J32_HR5). We made two deviations from this: the elephants were tested in four sessions instead of two, and we modified Smet and Byrne’s ^19^ ethogram.

### Ethics declaration

This study was approved by the National Research Council of Thailand (Protocol #0401/95). Ethical approval was obtained from the Faculty of Veterinary Medicine’s Animal Care and Use Committee (Protocol #R23/2566) at Chiang Mai University and the Wildlife Research Center’s Ethical Committee (Protocol #WRC-2023-009A) at Kyoto University. All methods were performed in accordance with the relevant guidelines and regulations, and this study is reported in accordance with ARRIVE guidelines. The elephants’ participation was voluntary, and the mahout (caretaker) could stop the experiment at any time if he felt the elephant did not want to participate anymore, but this never happened.

### Participants

Ten captive female Asian elephants (*Elephas maximus*) aged 11–61 (*M* = 36.8, *SD* = 17.16) from the Golden Triangle Asian Elephant Foundation living on the properties of the Anantara Golden Triangle Elephant Camp and Resort in Chiang Rai, Thailand, participated in the experiment between February and March 2024 (see Table S3).

### Experimental design

Each elephant was tested in four sessions conducted on separate days using a within-subjects, repeated-measures design. There was a 2-day break between sessions 1 and 2, a 6-day break between sessions 2 and 3, and a 2-day break between sessions 3 and 4.

**Fig. 2:**
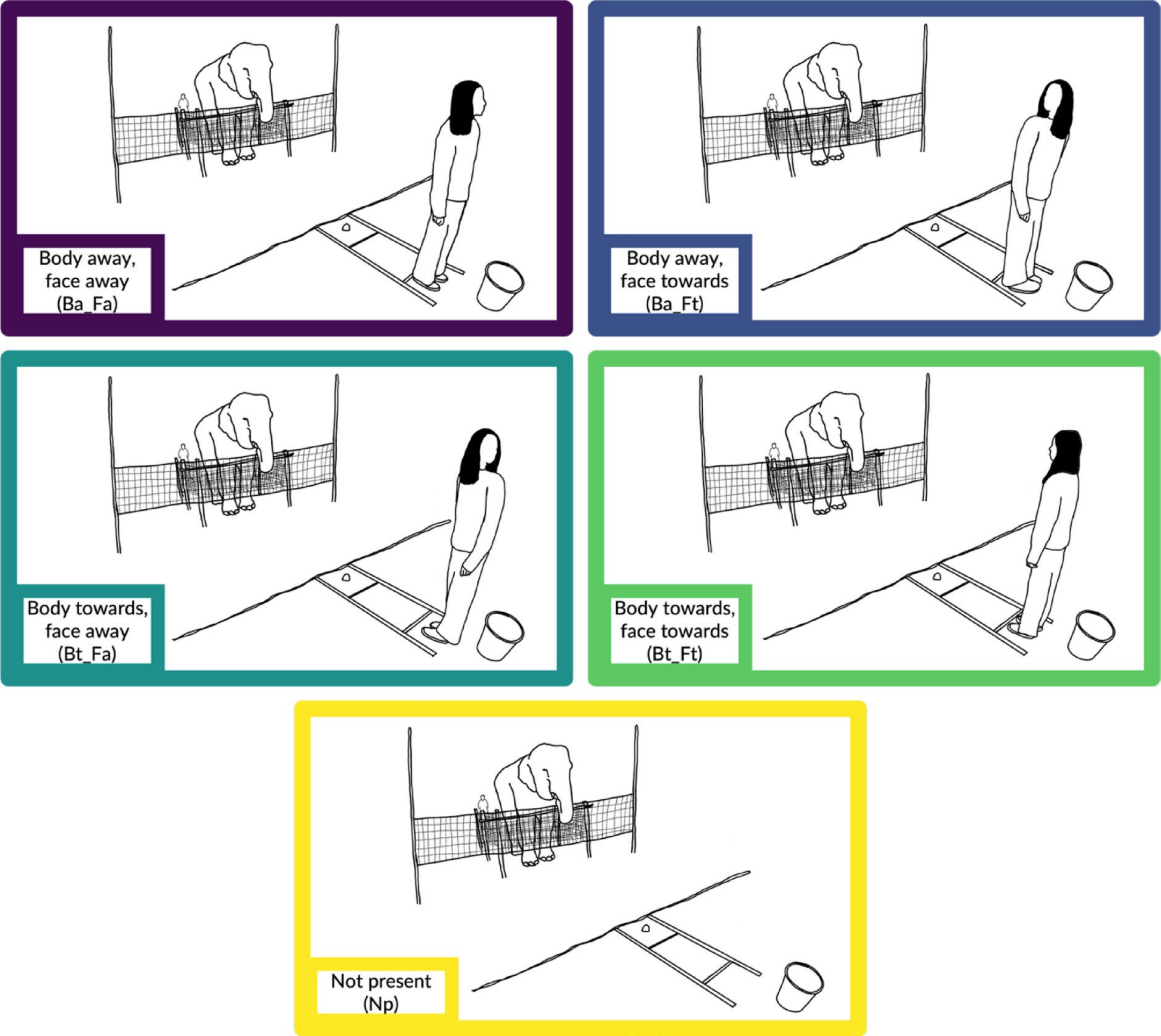
Experimental conditions showing the different orientations of the experimenter’s body and head, and the baseline condition. Illustrations by Hoi-Lam Jim.

In each session, the elephant completed one trial of each of the following conditions, which varied the orientation of the experimenter’s body and head, in a randomised order (Fig. 2):

1. Body away, face away (Ba_Fa)
2. Body away, face towards (Ba_Ft)
3. Body towards, face away (Bt_Fa)
4. Body towards, face towards (Bt_Ft)
5. Not present (Np)—henceforth ‘baseline’

We did not include the ‘body sideways’ conditions tested in Smet and Byrne ^19^ due to time constraints.

### Experimental setup

The experiment was conducted in a large field at the Anantara Golden Triangle Elephant Camp and Resort. The testing area (10.6 m [W] × 21.5 m [L]) was mowed, with tall grass surrounding the perimeter. A volleyball net (4.7 m [W] × 1.2 m [H]) was strung across the centre of the field, and a holding pen (2 m [W] × 3.7 m [L]) made of bamboo was built 35 cm behind the net to position the elephants during the experiment. The front and sides of the holding pen were covered with a volleyball net, while the back was left open so the elephant could exit freely. The experimenter (H.-L.J.; ‘E’) used a wooden tray (50 cm × 50 cm, with 1 m long handles) to feed the elephant during the experiment.

**Fig. 3:**
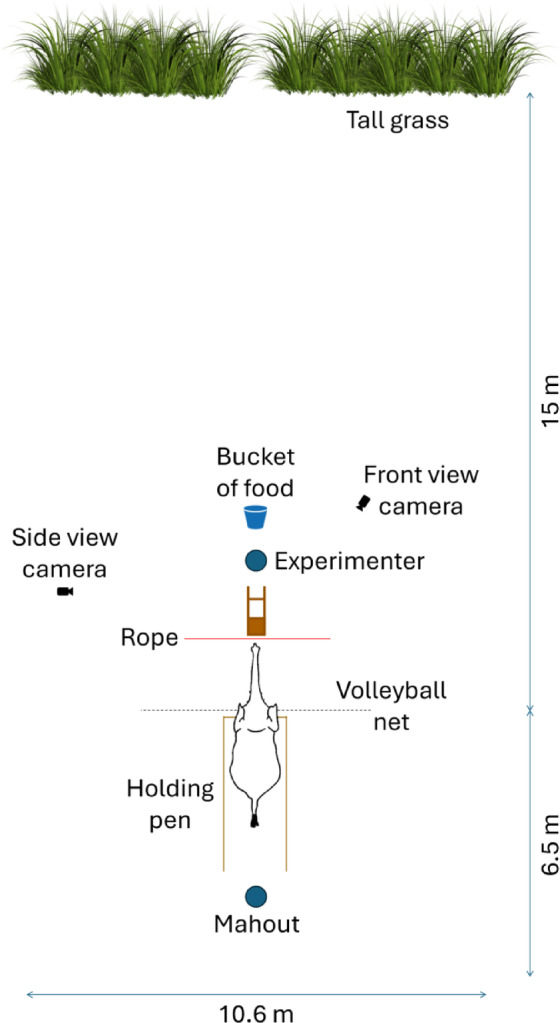
Schematic depiction of the experimental setup.

The whole experiment was recorded by two GoPro Hero 10 Black cameras. The side view camera was placed on a tripod at the side of the testing area and the front view camera was placed on a tripod close to E, facing the participant (Fig. 3).

### Procedure

Wild grasses were available ad libitum outside experiment times, and the elephants were additionally fed according to their regular feeding regimes; thus, they were not food-deprived before the experiment. Mangoes, a high-value food reward for elephants, were cut into half-pieces and used as the food reward. Each elephant was accompanied by her own mahout, who stood behind her during the experiment to avoid influencing her behaviour. However, there was one exception to this: due to the potential danger of working with one elephant (Yuki), her mahout wore sunglasses and initially stood to the side of her with his back towards E, so he was unaware of E’s posture. Once the mahout felt that Yuki was comfortable and the situation was safe, he moved to stand behind her. Throughout the experiment, the mahout only spoke to give commands, such as instructing the elephant to take the food if she did not reach for it when offered, or to stop if she physically interacted with the holding pen or volleyball net, or attempted to walk around the pen to retrieve the food.

Prior to testing, the elephant could explore the environment freely for approximately five minutes to familiarise herself with the location and the holding pen. Each mahout tested how far his elephant could reach with her trunk inside the holding pen, which ranged from 2.35–3.5 m, and the distance was marked by placing a rope on the ground. The tray was placed behind the rope; thus, the elephant supposedly could not reach the food. However, there were two occasions where the elephant managed to retrieve the food herself: one elephant (Jathong) pushed forward inside the holding pen, grabbed the tray, and pulled it towards herself; another elephant (Bo) retreated from the holding pen and walked around the volleyball net to eat from the tray and her mahout could not stop her in time. Both incidents occurred during the baseline after the test trial ended, thus the data were not excluded from analysis.

The experiment generally followed the procedure outlined in Smet and Byrne ^19^. All elephants were tested individually between 7:30 and 9 am or 2 and 3 pm depending on their availability, and each session took approximately 10 min. A session began with three ‘no-delay’ trials: E stood behind a wooden tray, called the elephant’s name whilst facing her, and placed a piece of food onto the tray. E then immediately picked up the tray and moved forward to allow the elephant to eat from the tray. After the elephant took the food, E placed the tray down in its original position. If the elephant did not take the food from the tray voluntarily in the first ‘no-delay’ trial because she was afraid of touching the volleyball net, the mahout showed the elephant the food and encouraged her to take it from the tray, and then another ‘no-delay’ trial was conducted to ensure the elephant was comfortable with taking the food from the tray by herself. Elephants also had additional ‘no-delay’ trials during the experiment if a brief interruption or minor experimental mistake occurred. After three consecutive ‘no-delay’ trials, the testing phase began with the first test trial.

In the test trial, E stood behind the tray, called the elephant’s name, placed the food on the tray, picked it up to show it to the elephant, and put it down without giving the food. Then, E adopted one of the four postures (or walked away in the baseline), started the stopwatch, and stood still for 20 s (i.e., the test trial period) before picking the tray up again and moving forward to feed the participant. In the baseline, E started the stopwatch as she turned to walk quickly to the tall grass and hid behind it; thus, she was obscured from the elephant’s view and ‘not present’. After 20 s passed, E walked back to her starting position and moved the tray within the elephant’s reach (see Video S2). Each test trial alternated with a ‘no-delay’ trial and sessions always ended with a ‘no-delay’ trial (see Fig. S1 for a flowchart of the procedure).

### Behavioural analysis

We coded the elephants’ actions towards E and the food during the test trial from the footage from the camera facing the elephant, and we supplemented it with the side view video footage when necessary.

We coded the frequency of head and trunk gestures produced to request food based on Smet and Byrne’s ^19^ ethogram. Some modifications were made because the elephants performed trained begging behaviours not observed in the previous study, and we refined the definitions by introducing subcodes (Table 1). Each behaviour was coded as a single event (i.e., if a behaviour was performed three times consecutively, it was coded as three separate behaviours).

**Table 1:** Definitions of coded behaviours.

Behaviour	Definition
Trunk swing	Tossing the trunk in the direction of E and/or the food in a quick, swift motion.
Trunk out	Slowly moving or holding some part of the trunk in the direction of E and/or the food. There were three subcodes based on the direction of the trunk tip: (1) forward (towards E); (2) self (towards the trunk or body); and (3) ground (towards the food).
Trunk up	Trunk upwards in an S-shape or holding some part of the trunk high above the head. There were three subcodes based on the direction of the trunk tip: (1) forward (towards E); (2) self (towards or touching the trunk); and (3) to the side.
Reach out	Full extension of the trunk. There were two subcodes based on where the trunk was pointing towards: (1) E or (2) the food.
Head nod	Head bobbing up and down at least once. One bout was coded as one event.
Trained begging	An “unnatural” behaviour taught by a human that is consistently performed on a verbal command. Only applicable to two elephants who performed distinct behaviours (Bo nodded her head and vocalised simultaneously, and Benz held her trunk out towards E, closed the airway to her trunk by holding the tip tightly together, then released it, creating a soft, suction-like sound).

### Statistical analysis

All statistical analyses were conducted using R (v4.4.1, ^36^) in RStudio (v2024.04.2 + 764, ^37^). The alpha level was set at 0.05 for all statistical tests. H.-L.J. and a research assistant independently coded 20% of the videos (10% from sessions 1 and 2 and 10% from sessions 3 and 4), which were randomly selected. Both coders were blind to the condition in each test trial. Interobserver reliability was assessed using the Intraclass Correlation Coefficient (ICC) from the *irr* package (v0.84.1, ^38^), which showed good inter-rater agreement ^39^: ICC (two-way, agreement) = 0.88, *F* = 15.4, *p* < 0.001. H.-L.J. coded the remaining videos blind to the condition in each trial.

Following the pre-registered analysis plan, we fitted a Generalised Linear Mixed-Effects Model (GLMM) using the *glmmTMB* package (v1.1.9, ^40^). Due to limited data for individual behaviours, we combined all behaviours and used the total frequency of head and trunk gestures in response to E’s visual attention as the response variable. The test predictor was ‘condition’ (a factor with five levels), with ‘session’ and ‘trial’ included as z-transformed continuous covariates. Participant ID was included as a random effect. The dataset comprised 200 observations from 10 individuals, with 92 instances of no recorded behaviours. As 46% of the data were zeros, we applied a zero-inflated Poisson GLMM.

To avoid multiple testing ^41^ and maintain type I error rate at the nominal level of 0.05, we compared the full model with a null model lacking the test predictor (‘condition’) using a likelihood ratio test (*anova* function with *method* = “*Chisq”*; ^42^). Pairwise comparisons with Tukey correction were conducted using the *emmeans* package (v1.10.2 ^43^). The plot (Fig. 1) was generated using *ggplot2* (v3.5.1 ^44^), with significance brackets added using *ggsignif* (v0.6.4 ^45^) and colours adjusted using *viridis* (v0.6.5 ^46^).

We assessed model diagnostics as follows: collinearity was evaluated by computing Variance Inflation Factors (VIF) ^47^ for a standard linear model using the *vif* function in the *car* package (v3.1.2, ^48^), which indicated no issues (maximum VIF = 1.014). Model stability was examined by comparing estimates from the full model with those obtained by systematically excluding each level of the random effect, one at a time. This indicated the model to be stable (see Table S1, for the estimate ranges). Confidence intervals were computed using the *boot.glmmTMB* function. The model was not overdispersed (dispersion parameter = 1.147). R functions used for assessing model stability, confidence intervals and overdispersion were provided by Mundry ^49^.

## Supplementary Information

Below is the link to the electronic supplementary material.

Supplementary Material 1

Supplementary Material 2

## Data Availability

The data and code associated with this manuscript are available in the Zenodo repository, 10.5281/zenodo.15486401.
