# Supplementary material for: Asian elephants (Elephas maximus) recognise human visual attention from body and face orientation
Source: Sci Rep. 2025 Oct 2;15:32623. doi: 10.1038/s41598-025-16994-3 (PMC12491582; doi:10.1038/s41598-025-16994-3)
Supplement: Supplementary file 1 — Supplementary Material 1 [file 41598_2025_16994_MOESM1_ESM.pdf]

Supplementary Information for:

Asian elephants (*Elephas maximus*) recognise human visual attention from body and face orientation

Hoi-Lam Jim[1,2*], Shinya Yamamoto[1], Pakkanut Bansiddhi[3,4], Joshua M. Plotnik[5,6*]

[1]Institute for the Future of Human Society, Kyoto University, Kyoto, Japan

[2]Japan Society for the Promotion of Science, Tokyo, Japan

[3]Faculty of Veterinary Medicine, Chiang Mai University, Chiang Mai, Thailand

[4]Center of Elephant and Wildlife Health, Chiang Mai University Animal Hospital, Chiang Mai, Thailand

[5]Department of Psychology, Hunter College, City University of New York, New York, USA

[6]Department of Psychology, The Graduate Center, City University of New York, New York, USA

*** Correspondence**

E-mail: jim.hoilam.6k@kyoto-u.ac.jp (H.-L.J.)

E-mail: joshua.plotnik@gmail.com (J.M.P.)

Author ORCID iDs

H.-L.J.: 0000-0002-8590-7167

S.Y.: 0000-0002-7556-6151

P.B.: 0000-0001-6699-4819

J.M.P.: 0000-0002-7597-8818

Table S1. Results from the zero-inflated Poisson GLMM predicting the frequency of head and trunk gestures. Abbreviations: Ba_Fa = Body away, face away; Ba_Ft = Body away, face towards; Bt_Fa = Body towards, face away; Bt_Ft = Body towards, face towards; Np = Not present.

Part[1]	Term	Estimate	SE	95% CI		Model stability		z	df	p[2]
				Lower	Upper	Min	Max			
Count	Intercept	-0.816	0.329	-1.567	-0.286	-1.098	-0.705			
	Condition: Ba_Fa[3]	0.658	0.271	0.175	1.247	0.539	0.739	2.427	4	0.015
	Condition: Ba_Ft	0.728	0.272	0.223	1.372	0.632	0.874	2.672		0.008
	Condition: Bt_Fa	0.938	0.262	0.412	0.543	0.804	1.059	3.587		< 0.001
	Condition: Bt_Ft	1.282	0.249	0.828	1.901	1.128	1.444	5.152		< 0.001
	Session[4]	-0.318	0.078	-0.467	-0.187	-0.392	-0.251	-4.078	1	< 0.001
	Trial[4]	0.018	0.068	-0.129	0.163	-0.017	0.055	0.265	1	0.791
Zero	Intercept	-2.433	0.776	-20.342	-1.543	-4.212	-2.183			

Estimate, standard errors (SE), 95% confidence intervals (CI), model stability (estimate ranges derived after excluding individuals one at a time) and results of significance tests (Wald's *z* approximation).

[1]'Count' indicates the count part and 'Zero' the zero-inflation part of the model

[2]The *p* value for the intercept is not shown due to its limited interpretability

[3]Reference level for Condition = Np

[4]Continuous variables (Session and Trial) were z-transformed (Session: *M* = 2.5, *SD* = 1.121; Trial: *M* = 3, *SD* = 1.418)

Table S2. Pairwise comparisons. Significant *p* values are in bold. Abbreviations: Ba_Fa = Body away, face away; Ba_Ft = Body away, face towards; Bt_Fa = Body towards, face away; Bt_Ft = Body towards, face towards; Np = Not present.

Comparisons	Estimate	SE	*p*	95% CI	
				Lower	Upper
Np – Ba_Fa	-0.658	0.271	0.108	-1.397	0.082
Np – Ba_Ft	-0.728	0.272	0.058	-1.471	0.015
Np – Bt_Fa	-0.938	0.262	**0.003**	-1.651	-0.225
Np – Bt_Ft	-1.282	0.249	**< 0.001**	-1.961	-0.603
Ba_Fa – Ba_Ft	-0.070	0.226	0.998	-0.687	0.548
Ba_Fa – Bt_Fa	-0.280	0.211	0.674	-0.856	0.296
Ba_Fa – Bt_Ft	-0.625	0.197	**0.013**	-1.162	-0.087
Ba_Ft – Bt_Fa	-0.210	0.214	0.864	-0.795	0.374
Ba_Ft – Bt_Ft	-0.555	0.199	**0.042**	-1.097	-0.012
Bt_Fa – Bt_Ft	-0.344	0.184	0.335	-0.847	0.158

Table S3. Individual characteristics of participants.

Elephant	Age (years)	Experience in other experiments
Bo	46	Yes – prior
Benz	18	Yes – first time simultaneously
Boonma	61	No
Boonrod	29	Yes – first time simultaneously
Boonsri	56	Yes – prior
Dah	22	Yes – prior
Jathong	33	Yes – prior
Kummool	54	Yes – prior
Yokfah	11	Yes – first time simultaneously
Yuki	38	Yes – prior

Video 1. Example of a test trial in the 'Body towards, face towards' condition.

https://youtu.be/6c3mXxT_SDA

Fig. S1. Flowchart illustrating an example of the full procedure for one participant. Colours correspond to the experimental conditions shown in figures 2 and 3 of the main text. Abbreviations: Ba_Fa = Body away, face away; Ba_Ft = Body away, face towards; Bt_Fa = Body towards, face away; Bt_Ft = Body towards, face towards; Np = Not present.

Session 1	2-day break	Session 2	6-day break	Session 3	2-day break	Session 4
1. No-delay trial		1. No-delay trial		1. No-delay trial		1. No-delay trial
2. No-delay trial		2. No-delay trial		2. No-delay trial		2. No-delay trial
3. No-delay trial		3. No-delay trial		3. No-delay trial		3. No-delay trial
4. Test trial 1 (Ba_Fa)		4. Test trial 1 (Np)		4. Test trial 1 (Bt_Fa)		4. Test trial 1 (Ba_Ft)
5. No-delay trial		5. No-delay trial		5. No-delay trial		5. No-delay trial
6. Test trial 2 (Bt_Fa)		6. Test trial 2 (Bt_Ft)		6. Test trial 2 (Ba_Fa)		6. Test trial 2 (Np)
7. No-delay trial		7. No-delay trial		7. No-delay trial		7. No-delay trial
8. Test trial 3 (Ba_Ft)		8. Test trial 3 (Ba_Fa)		8. Test trial 3 (Ba_Ft)		8. Test trial 3 (Bt_Ft)
9. No-delay trial		9. No-delay trial		9. No-delay trial		9. No-delay trial
10. Test trial 4 (Np)		10. Test trial 4 (Bt_Fa)		10. Test trial 4 (Bt_Ft)		10. Test trial 4 (Ba_Fa)
11. No-delay trial		11. No-delay trial		11. No-delay trial		11. No-delay trial
12. Test trial 5 (Bt_Ft)		12. Test trial 5 (Ba_Ft)		12. Test trial 5 (Np)		12. Test trial 5 (Bt_Fa)
13. No-delay trial		13. No-delay trial		13. No-delay trial		13. No-delay trial
